# Enhancing diagnostics through flow cytometry: overcoming barriers in low resource settings

**DOI:** 10.3389/fonc.2025.1604295

**Published:** 2025-09-12

**Authors:** Jeanine Alfaro, Patricia Valverde, Paola Salguero, Xiomara Arevalo, Pamela Ortega, Luisa Sanchez, Federico Antillon-Klussmann, Randall T. Hayden, Alicia Rodriguez, Monika L. Metzger, John K. Choi, Victor M. Santana, Paul E. Mead, Priya Kumar

**Affiliations:** ^1^ Department of Pediatrics, Unidad de Oncología Pediátrica, Guatemala City, Guatemala; ^2^ Francisco Marroquin University, School of Medicine, Guatemala City, Guatemala; ^3^ Department of Pathology, St. Jude Children’s Research Hospital, Memphis, TN, United States; ^4^ Department of Global Pediatric Medicine, St. Jude Children’s Research Hospital, Memphis, TN, United States; ^5^ Department of Pediatrics, University of Tennessee Health Science Center, Memphis, TN, United States

**Keywords:** low- and middle-income countries, flow cytometry, minimal residual disease, pediatric, acute lymphoblastic leukemia

## Abstract

Despite advancements in the treatment of acute lymphoblastic leukemia (ALL), survival rates vary greatly based on factors such as cancer subtype and geographical location. The use of diagnostic tools like immunophenotyping and measurable residual disease (MRD) detection by flow cytometry (FC) are key to optimal management. FC offers higher sensitivity and rapid turnaround compared to traditional methods like morphological evaluation and immunohistochemistry. However, in low- and middle-income countries (LMIC), the adoption of robust MRD assays via FC remains limited due to challenges such as technical complexity, the need for operator expertise, and the high costs associated with antibodies and quality control. This study discusses the design, implementation, and validation of MRD strategies using FC in resource-constrained environments, highlighting the potential for improved accessibility and patient outcomes when these barriers are addressed.

## Introduction

Quality diagnostics are crucial to improving health care outcomes (1). Flow Cytometry (FC) is essential for leukemia diagnosis and follow-up (2). However, its implementation in low- and middle-income countries (LMICs) faces challenges across all testing phases: pre-analytic, analytic and post-analytic (3, 4). These barriers include but are not limited to an unreliable supply chain of quality fluorochrome-labeled antibodies, challenges in reliable transportation of samples, limited availability of experts trained in FC data interpretation and challenges in obtaining samples for validation of assays and underutilized machinery (5, 6). Evidence also suggests that quality of care is hindered by underrepresentation of complex or specialty-specific cases resulting in knowledge gaps and suboptimal patient management (7). There are several steps required for processing a sample by FC, each requiring a contextually adapted solution. Herein we describe the optimized testing at a single, standalone FC laboratory, operating in a resource constrained setting, where a simplified FC assay was implemented creating both an effective and sustainable impact on healthcare. Implicit in these considerations are potential lessons for future stepwise implementation of high complexity FC assays in other resource-limited settings.

## Pre-analytic barriers

Optimal management of childhood acute lymphoblastic leukemia (ALL) in high resource countries includes the use of diagnostic tests, such as immunophenotyping by FC and assessment of minimal residual disease (MRD) by FC (8, 9). FC is widely available in LMICs due to its prior use in HIV treatment. However, having a flow cytometer does not guarantee quality diagnostics. Training and technical expertise are essential for clinical use (10).

### Setting

The Lorenzana FC laboratory in Guatemala initially focused on HIV diagnostics but expanded to leukemia diagnostics in 1998. Between 1998 and 2016, 7,916 suspected pediatric leukemia cases were processed, averaging 416 per year (11). Diagnostic case frequencies are represented in **Supplementary Figure 1a**. A partnership with St. Jude Global in 2016 enabled further assay optimization, without sacrificing diagnostic accuracy (**Supplementary Figure 1b**). While this assay only utilized 1 of the 2 available lasers, it was optimal for initial adoption of leukemia diagnostics. Analysis of three antibodies at a time is technically easier and allows for dichotomous logic during analysis (**Supplementary Table 1**). In 2019, a simplified MRD assay was implemented, increasing capacity and efficiency (**Supplementary Table 2**).

### Sponsorship

Leadership support is one of the most critical factors for implementation and integration of a fully accessible FC diagnostics workflow (12). Departmental vision and allocation of resources is heavily dependent on enterprise and departmental leadership. This authoritative backing is crucial for the implementation and sustainability of a clinical care delivery model that includes FC at diagnostic and follow up time points. One of the most highly cited barriers to full adoption is lack of executive leadership buy-in (13–15). Careful incremental expansion is a suggested strategy to gain leadership support, as the adoption of new assays requires added resources and champions who are aware of the risks involved in rapid expansion. In this case the Lorenzana FC laboratory implemented a stepwise approach starting with refitting their three-color panel to four colors to fully utilize their machine for leukemia lineage assignment. This resulted in less waste and more efficient usage, with the laboratory being able to perform more tests in the same amount of time.

### Supply-chain

**Figure 1:**
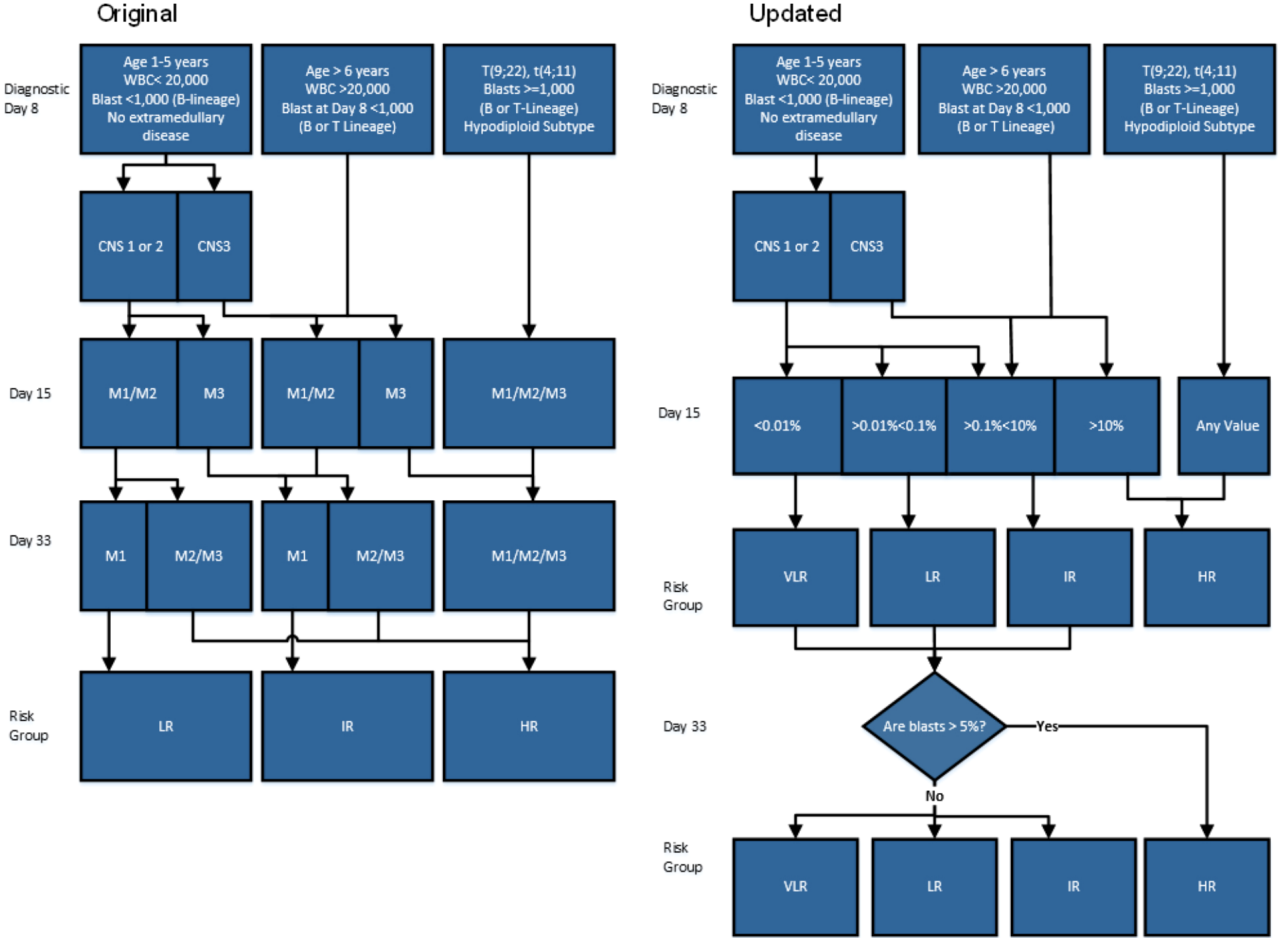
Comparison of two work flows titled Original and Updated illustrating leukemia risk stratification based on diagnostics. **(a)** The original version represents the workflow prior to MRDLite implementation. **(b)** The Updated version represents workflow post MRDLite implementation Legend: WBC, White Blood Cells CNS, central nervous system; CNS 1, cerebrospinal fluid (CSF) without blasts and nontraumatic; CNS 2, CSF ≤ 5 cells/mm^3^ with blasts, or the spinal tap was traumatic (>10 erythrocytes/mm^3^) or was performed > 72 hours after the beginning of therapy; CNS 3, CSF > 5 cells/mm^3^ with blasts, or cerebral nerve palsy, or a cerebral mass; M1, < 5% BM blasts; M2, 5% to 24% BM blasts; M3, ≥ 25% BM blasts; VLR, Very Low Risk; LR, Low Risk; IR, Intermediate risk; HR, High Risk. Day 33 Marrow: “Are blasts >5%” refers to Bone marrow morphologic blast estimate.

Use of any antibodies for clinical FC in Guatemala requires each antibody-fluorochrome conjugate to be registered and approved by the Ministry of Health. This process takes approximately six months to complete through standard channels and can be delayed for various reasons. In our regional setting, the first step in initiative regulatory approval begins with a request to the manufacturing company, who must internally approve whether to allocate resources for the process. This initial step may take several months and must be completed at least a year in advance of purchase. Regulatory approval requires specific documentation for each reagent, including reason for use, technical and safety data translated into the local language. This documentation must be provided by the manufacturing company directly to the local regulatory body, in this case the Ministry of Health. It is necessary to engage in advocacy with the manufacturing company to obtain their willingness to carry out this process; otherwise, the institution must assume the costs of the entire submission, which may be prohibitive. Successful implementation and validation of the new diagnostic panel enabled processing of 551 newly diagnosed cases in the year 2019 (**Figure 1b**), compared to a capacity of 439 cases per year between 1998 and 2016. Furthermore, approximately 369 patients were eligible for ALL specific treatment with a confirmed diagnosis, compared to 112 patients per year between 2007 - 2014 (11). This increase in access resulted in additional funding to buy new reagents, hire and train new personnel for hours allocated for minimal residual disease assay implementation. To implement an MRD approach with the sensitivity needed for risk stratification, an upgraded flow cytometer was needed, upgrading from a BD FACS Calibur (2 lasers, 4 colors) to the BD FACS Lyric with (3 lasers, 8 colors).

### Space

Space allocation in the laboratory has traditionally been challenging. There are many steps involved in receiving FC specimens and processing, including use of antibodies that are light sensitive, making window placement particularly important. Optimal placement of the flow cytometer is essential to provide a low traffic, clean and chemical and spill-free location with minimal floor vibration, away from specimen mixers. A careful plan to optimize placement of sinks, staining benches and a flow cytometer is essential to improve efficiency, maintain quality turnaround time and improve job satisfaction of the laboratory staff. An ideal laboratory should have dedicated clean and dirty zones, with an open workspace arrangement, and linear workflow. Prior to the remodeling, there were no properly defined clean and dirty areas. The areas were undivided, and access from the laboratory to the offices required walking through the sample processing area. The reagent preparation and staining areas were adjacent to the office area. Therefore, there was no adequate workflow or proper separation between the areas. With support from leadership the Lorenzana clinical laboratory upgraded their space to be more efficient requiring the same amount of physical space and incorporated greenery and relaxation spots (**Supplementary Figure 2**).

## Methods

### Resource adapted implementation

After implementation of leukemia diagnostics, the next goal is to risk stratify patients. Few centers can risk stratify based on genomic subtypes in a clinically relevant time frame. Another accessible approach to stratify patients in addition to age, white blood cells count (WBC) and lineage subtype is detection of minimal residual disease by FC up to day 15 after the start of induction therapy. At St. Jude Children’s Research Hospital (SJCRH) a simplified MRD assay (MRDLite) was developed and validated, using only four antibodies with sufficient predictive power to identify measurable residual disease at time points prior to hematogone regeneration (16, 17). Since the registration of new antibody-fluorochrome conjugations was cumbersome, the panel was designed in a way to minimize any switching of fluorochrome-antibody conjugates and only added CD10 to the APC channel (**Supplementary Table 1**). MRDLite is only useful at Day 15 and for patients with suitable immunophenotype at diagnosis, meaning those that are both CD19 positive and CD10 positive or both CD19 and CD34 positive. At a similar site in Recife, Brazil, by incorporating MRDLite into their risk stratification protocol they were able to identify patients at very low-risk assigning them to a reduced intensity antimetabolite based treatment protocol (17). To synergize with the Unidad de Oncología Pediátrica (UNOP)’s desire to introduce MRDLite into its therapeutic decision making, we aimed to establish the effectiveness of the new assay design in the context of the local treatment regimen. Data were collected from 2019 to 2021 from patients aged 1 to 17 years with newly diagnosed ALL. Diagnosis of ALL was based on morphologic assessment of modified Giemsa-stained smears of blood or bone marrow. The diagnostic FC panel included TdT, cCD3, CD45, CD19, MPO, CD34, cCD79a with additional add-ons based on results. DNA Index by FC and diagnostic RT-PCR panels were also utilized.

Samples were collected in EDTA tubes and processed within 24 hours of receipt in the laboratory. While the majority of samples submitted were fresh samples collected the same day, this was not always the case, since the laboratory is located five kilometers away from the main hospital and collection times were not always during normal business hours. All samples were reported within a maximum of 72 hours from time of collection. For assessment of sample viability, scatter properties were assessed and debris exclusion performed via a FSC *vs* SSC plot.

Risk Stratification Criteria – According to the acute lymphoblastic leukemia-Guatemala trial (LLAG - 077) protocol, B-cell precursor ALL patients were stratified into standard-risk, intermediate-risk and high-risk groups according to the criteria summarized in **Figure 1a**. Prednisone-poor response was classified by morphology and defined as having greater than or equal to 1000 blasts/mm (3) in peripheral blood after seven days of prednisone and one intrathecal dose of methotrexate. MRD levels were evaluated by FC at Day 15 and re-classified based on positivity according to criteria summarized in **Figure 1b** showing an updated patient risk assignment after MRD interpretation.

### Antibodies

The four-color antibody panel included antibodies against CD19, CD10, CD34 and CD45, obtained from BD Biosciences San Jose, CA, USA. All antibodies were appropriately titrated to optimize the staining and best separation between the negative and positive populations. The individual titers are indicated in **Supplementary Table 2**. For routine processing, an antibody cocktail for weekly use was prepared using reagents only, without buffers. They were stored in the fridge (2 - 8degrees C) in amber vials. Cocktails were stored for a period of one month (unless one of the reagents used had an earlier expiry date) and validated using internal controls (18–20).

### Cell preparation and staining protocol

Historically, Ficoll-separated samples were used to acquire mononuclear cells for flow cytometric analysis (21). This pre-analytical step is labor intensive. Bulk lysis is a technically easier process that provides a total WBC sample suitable for FC analysis. Genuardi et al, provided comparison data for MRD analysis using both total WBC and mononuclear cells recovered by bulk lysis and Ficoll methodology and reported similar results (22). To replicate its performance we looked at an initial group of 10 B-ALL cases at day 15 post induction chemotherapy. Each sample was divided, and cells were prepared using both bulk-lysis and Ficoll density gradient centrifugation. The first comparison between the two approaches demonstrated that bulk-lysis had higher cell counts compared to Ficoll density gradient centrifugation with a median total WBC (CD45 positive) recovered of 404,000 events *vs* 353,000 events. To validate the limit of detection we compared MRD calculations using the two red blood cell removal methods. For the bulk-lysis samples, we applied a limited scatter gate to eliminate neutrophils from the total gated events to mimic the loss of this population when samples are prepared using Ficoll density separation. MRD values for each method were compared with attention to those results close to the 0.1% cutoff for inclusion in the local standard risk versus high-risk protocol. All of these pair-wise samples correlated with an R-value close to 1 (0.999, p = 1x10^-9^) and (0.972, p = 5.6x10^-5^) for samples close (range 0.2% to 0.09%) to the 0.1% cutoff (see **Supplementary Figure 3**).

The cellularity of all samples was assessed morphologically based on cytospin evaluation. If there were increased blasts (greater than 5% in 100 cells counted), a lower number of events were collected. In the case of hemodiluted samples, a larger sample volume was assayed (200ul was used instead of 100ul for standard bone marrow aspirate samples). For markedly paucicellular samples, cells were concentrated by pelleting and reconstituting them to 200ul of PBS before staining and post staining lysis.

### Acquisition and analysis

Samples were acquired on a BD FACS Calibur flow cytometer platform using BD Cellquest software version 8 for acquisition and analysis. The instrument set up and daily quality control was performed using CS&T beads (BD Biosciences). For each sample, up to 500,000 events were acquired and analyzed.

### MRD calculation and detection limits

MRD positivity was identified based on a cluster showing either CD19 and CD10 positivity or CD19 and CD34 positivity. MRD results were given as percentages of mononuclear cells calculated from a “pseudo-Ficoll” gate. A correction factor was determined using the “mononuclear cells” as determined by the pseudo-Ficoll gate while subtracting the Syto Negative CD45 negative events from the calculation. These numbers were gated and calculated using Tube 2 detailed in **Supplementary Table 2 (** 23, 24). We used a limit of detection of ten events that formed a cluster, and fell outside of the debris field using a “back gating” strategy.

## Impact

**Figure 2:**
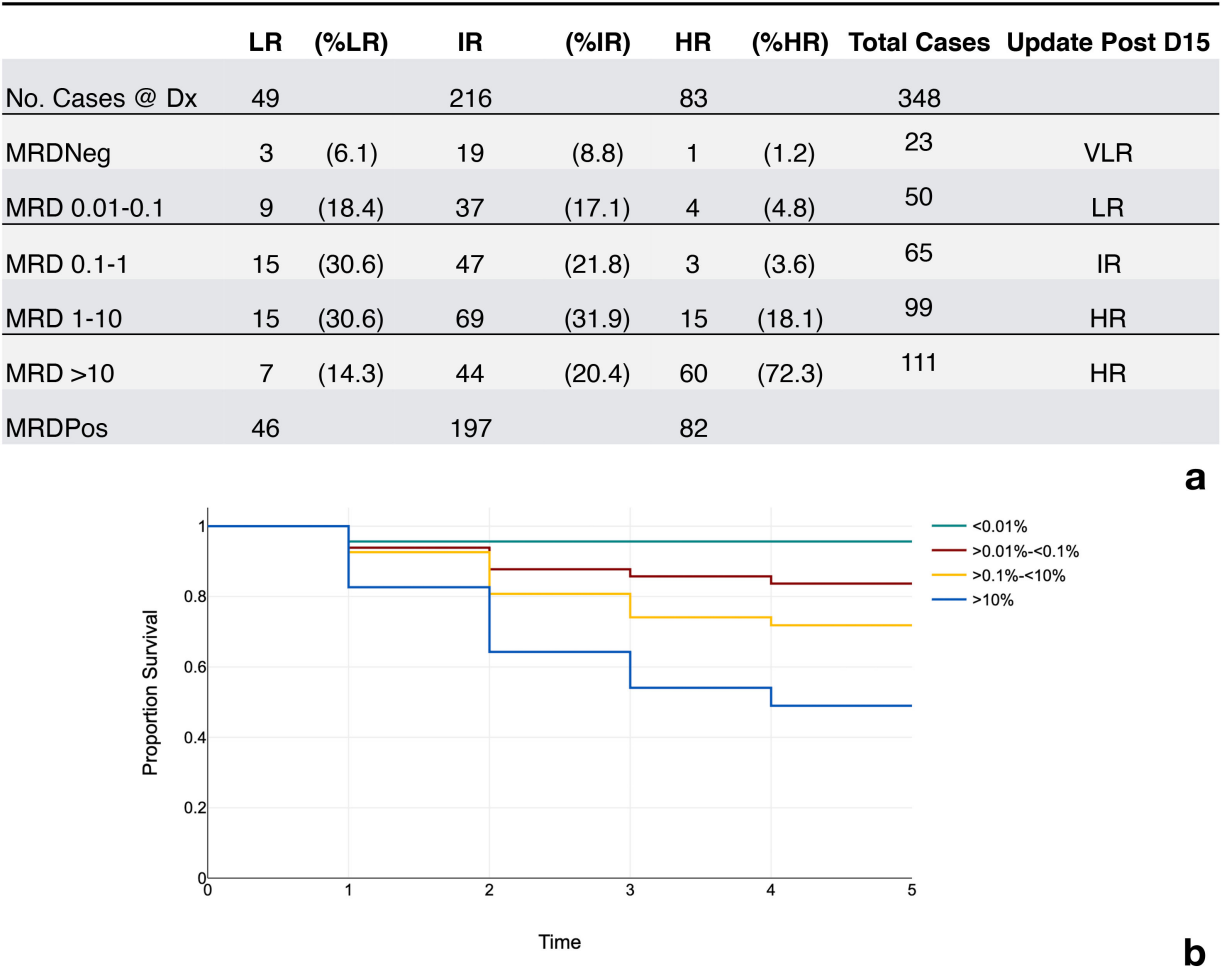
Incidence of MRD results and event free survival rates. **(a)** Incidence of MRD positivity and respective risk categories. LR, low risk; IR, intermediate rise; HR, high risk; VLR, very low risk; MRDNeg (<0.01% of the mononuclear cells), MRDPos (<0.01% of the mononuclear cells.) MRD Levels in column 1 represent percent of the mononuclear cells using a correction factor. **(b)** Rate of event free survival over time represented in years.

Between May 2019 and December 2021 the laboratory received a total of 360 samples for B cell MRDLite evaluation. All results were reported within 48 hours of receipt in the laboratory. We retrospectively studied B cell MRD cases across all age groups. All cases were pediatric patients and treated under the Unidad de Oncología Pediátrica leukemia protocol. Out of 360 samples, twelve samples were both CD10 and CD34 negative and, therefore, unsuitable for MRDLite analysis. Of the 348 remaining samples, 325 were positive on day 15, with 93.3% incidence at levels ranging from 0.61% to 36%. **Figure 2a** displays a detailed distribution of the 348 cases based on the level of MRD detected (<0.01%, 0.01 - 0.1%, 0.1 - 10%, and greater than 10%). Samples acquired using ACK bulk lysis processing method yielded a higher number of analyzable mononuclear events than Ficoll samples, 404,000 *vs* 353,000 events. A small validation of MRD results derived from split samples processed using Ficoll method and ACK Lysis method showed similar results (R = 0.99, p = 1x10^-11^). All samples morphologically positive by hematopathology review of bone marrow aspirates were positive by FC assessment of MRDLite. 82% of samples that were morphologically negative were positive by flow. A morphologic blast estimate from cytospin was performed at the flow lab and the rate of discrepancy between flow cytometry results >5% improved over time, while the rate of discrepancy by hematologist review stayed the same. **Figure 2a** displays results that are further subcategorized based on risk categories at day 15 according to previously published St. Jude Total trials (25). Taking this into account, 67 (19.2%) patients would have received less intensive therapy than their previous risk category. Alternatively, 150 (43%) would have received more intense therapy and been upgraded to either intermediate or high-risk categories. Of the patients treated under the Guatemalan front line ALL protocol, the incidence of relapse increased with greater levels of minimal residual disease as shown in **Figure 2b**. The MRD values were positively correlated with other clinical risk features like WBC count and age. Only one relapse was recorded in the MRD negative category. This relapse was a CNS only and represented a patient with t(1;19).

## Discussion

B-ALL is a leading pediatric malignancy, and MRD quantification is essential for guiding treatment (26). Our data demonstrate that MRD results obtained with a simplified FC approach, when combined with clinical features, can identify patients with low risk of relapses, as well as identify patients that are at higher risk of treatment failure. In this single center experience the rate of positivity on day 15 was 93.3%. The results reveal a higher proportion of day 15 positivity compared to that reported by two European centers following the AIEOP-BFM-ALL (MRD >0.01% 77.1% and MRD>0.1% 74.1, respectively) (27, 28). This may be due to several factors. The data described in the present study were collected during the COVID - 19 pandemic when supply chains and patient accessibility was limited. Other factors, including the use of bulk lysis methodology resulting in relatively higher event acquisition, could have played a role. Furthermore, there may be differences in the patient population, as in Guatemala there is an overrepresentation of higher risk molecular subtypes, for example CRLF2 rearranged cases of BALL (29).

While the clinical role of MRD by FC in the context of BALL is well known implementation is difficult especially when considering supply chain issues in resource constrained settings. While the regulatory process described here reflects the Guatemalan context, similar barriers exist globally. Institutions aiming to introduce new clinical reagents should begin by identifying their national regulatory authority, often a Ministry of Health or equivalent, and seek guidance from local laboratories or professional societies with experience in clinical assay approvals. Diagnostic manufacturers can also provide country-specific regulatory pathways and documentation requirements. Early engagement with these stakeholders is essential, as approval timelines may be lengthy. Our experience highlights the importance of proactive communication and advocacy with both regulatory bodies and suppliers to ensure sustainable access to critical reagents.

One practical concern during day 15 bone marrow analysis is hemodilution or poor sample quality which can lead to false low level or false negative MRD values. While increasing the number of acquired events helped in part, the introduction of SYTO13 and CD235a antibodies to calculate the percentage of mononuclear cells proved helpful to normalize the denominator. For example, if the calculation factor is higher than five, this would trigger an inclusion criteria for a limited sensitivity disclaimer on the report and recommend re-draw if clinically indicated. This provides a standard process and increased communication between the laboratory and clinical team.

Lack of standardization of MRD assays is a major challenge (30). While this is a simplified assay, the enumeration of MRD remains critical to define clinically relevant cutoffs. Historically, total white cells or non-erythroid cells based on CD45-positive events have been commonly used as a denominator (21). Various studies are now using different methods to determine the denominator for MRD calculation (9, 21, 30, 31). Many studies have recommended an additional nuclear-binding dye (Syto13) tube for calculation of corrected MRD in total nucleated cells (21, 32, 33).

Several global sites around the world collaborate with different centers to seek advice on treatment (34). The Lorenzana laboratory incorporated markers like Syto13 and CD235a to facilitate cross-institutional comparison and increase clinician confidence in results. Providing contextual data allows for comparison of results and reaching conclusions to get relevant clinical advice, making this Guatemalan FC laboratory especially suited to act as a reference center for various different institutions in their region.

The results presented here were from studies performed during the COVID 19 pandemic, which has some bearing on the outcome data. For example, drug shortages including asparaginase were common during this period. Furthermore, clinically relevant follow up data was difficult to reliably gather during the peak of the pandemic and the travel restrictions that were manifest. These factors must be considered to ascertain the correlation between MRD positivity and survival outcomes that were statically significant or meaningful. The results presented here, however, confirmed that there is a low probability of relapse in the MRD negative population. The rate of positivity correlated well with other clinical risk factors like WBC count and age. The rate of relapse increased with MRD value. There was significant clinical relevance with respect to upgraded risk stratification, where 19% of patients would have been downgraded and 48% of patients upgraded.

While this simplified method is not applicable at later treatment time due to the presence of normal regenerating B cells (hematogones), the implementation, consistent performance and reporting of results in a clinically relevant time frame translated to increased trust in the results within their healthcare system, and therefore access to patients. Since the MRDLite assay became available, Guatemalan children have benefited from flow-based risk stratification that was not previously possible. The laboratory was able to reliably report within 48 hours of receipt greater than ninety percent of the time.

The introduction of cytospin analysis can help improve morphologic discrepancies in blast estimation, in part due to the immediate feedback from FC estimation. This gain in knowledge introduces an increase in expertise and specialization. In many centers around the world with limited resources, a hemato-oncologist may preview the aspirate smears and provide a differential, without formal hematopathology training. Running a FC laboratory or specializing in the FC analysis may provide a passive transfer of knowledge that includes morphologic training. At the beginning of our partnership, and outlined in an earlier publication, the amount of consultation with St. Jude decreased from 20% of their cases to just 3% in three years (10). Introducing bulk lysis costs about ten cents (USD) per sample versus $150 per sample using Ficoll samples. These cost data include reagents and time, which in total amounts to over $50,000 savings for 360 tests. These small optimization steps decreased costs and turnaround times while also improving operator confidence. All of these steps were integral in securing institutional support to upgrade from a two-laser to a three-laser flow cytometer. We emphasized the projected increase in testing capacity, reduced turnaround time, and the potential for improved patient risk stratification using MRDLite. Importantly, we linked these improvements to tangible clinical outcomes, such as enabling timely therapeutic decisions and increasing the number of patients eligible for protocol-based care. Our business case also highlighted cost savings from transitioning to bulk lysis (over ficoll), reductions in external consultation needs, and the lab’s growing role as a regional reference center. For other centers, we recommend collecting baseline metrics (e.g., test volume, failure rates, turnaround time) and aligning the proposal with institutional or public health goals. Framing the upgrade as an investment in both operational efficiency and patient impact helped build consensus among leadership and secure the necessary resources.

The program’s success is evident in its ability to train regional partners, doubling the number of trainees in five years. Standardized workflows and quality assurance programs contributed to workforce stability and knowledge retention. The adoption of bulk lysis processing reduced costs, making MRD assessment more accessible. The Lorenzana laboratory, throughout the years, has provided technical education for their regional partners. Pathologists and hemato-oncologists with special interest in FC also receive training. They provide technical assistance above and beyond that provided by the cytometer manufacturers. For example, their experience with their new more complex cytometer instruments helped inform a neighboring site in Honduras acquire and onboard a similar instrument. Brain-drain is a prevalent factor in low resource areas. The FC laboratory in Guatemala has enjoyed a steady and reliable workforce with a good mix of long-term, mid-term and short-term employees. This could be in part due to standardized workflows that are clear and developed internally with the support of external quality assurance programs, to reflect their current situation.

## Conclusion

This study demonstrates the feasibility and clinical value of implementing a simplified, resource-adapted flow cytometry-based MRD assay in a low-resource setting. Through a stepwise approach that addressed key barriers, including supply chain limitations, regulatory hurdles, spatial constraints, and lack of technical expertise, the Lorenzana laboratory successfully expanded diagnostic capacity, reduced turnaround times, and improved risk stratification for pediatric ALL patients (see **Supplementary Table 3**). The use of MRDLite, in combination with bulk lysis processing and internal quality checks, enabled timely therapeutic decision-making and cost-effective diagnostics. Moreover, the program fostered regional capacity building, institutional trust, and workforce stability. These findings offer a scalable blueprint for other resource-constrained settings seeking to integrate high-complexity diagnostics into routine care and underscore the importance of context-specific innovation in advancing global health equity.

## Data Availability

The original contributions presented in the study are included in the article/**Supplementary Material**. Further inquiries can be directed to the corresponding author.
