# Supplementary figures and images for: Enhancing diagnostics through flow cytometry: overcoming barriers in low resource settings
Source: Front Oncol. 2025 Sep 12;15:1604295. doi: 10.3389/fonc.2025.1604295 (PMC12463636; doi:10.3389/fonc.2025.1604295)

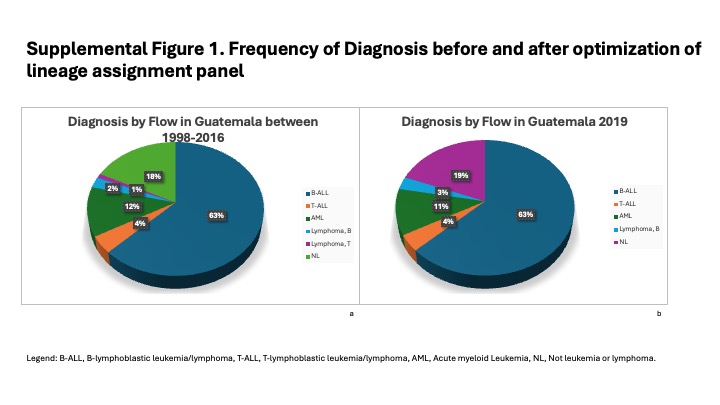

Supplement: Supplementary file 1 [file Image1.jpeg]

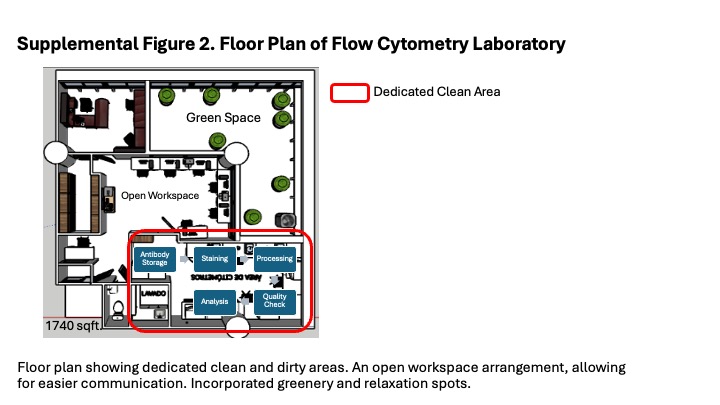

Supplement: Supplementary file 2 [file Image2.jpeg]

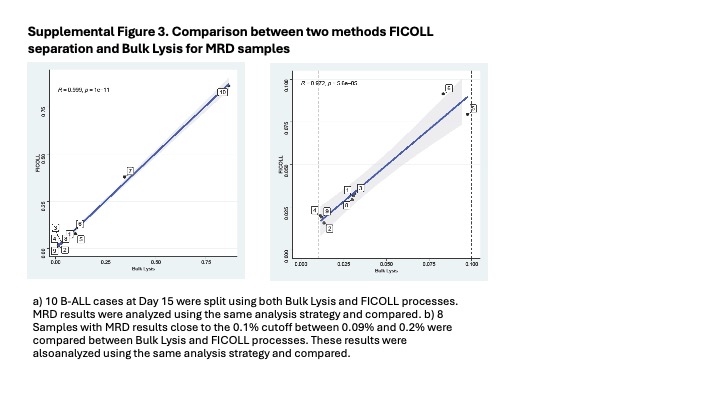

Supplement: Supplementary file 3 [file Image3.jpeg]

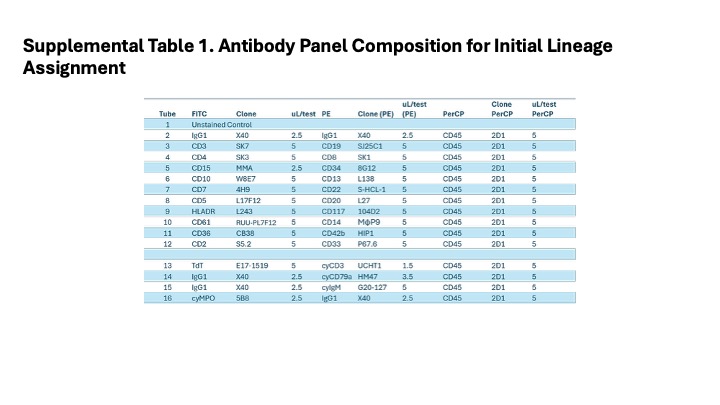

Supplement: Supplementary file 4 [file SupplementaryFile1.jpeg]

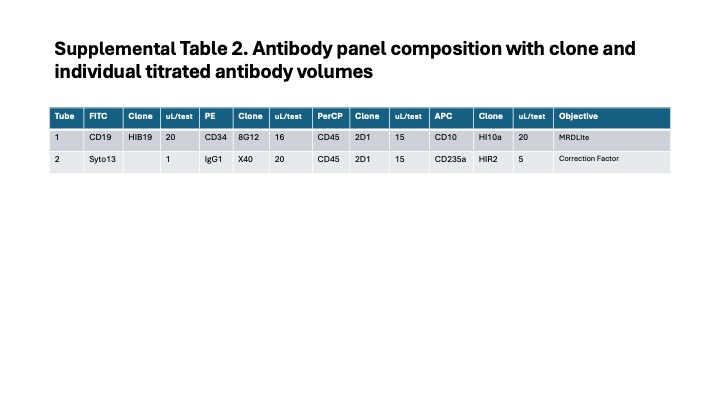

Supplement: Supplementary file 5 [file SupplementaryFile2.jpeg]

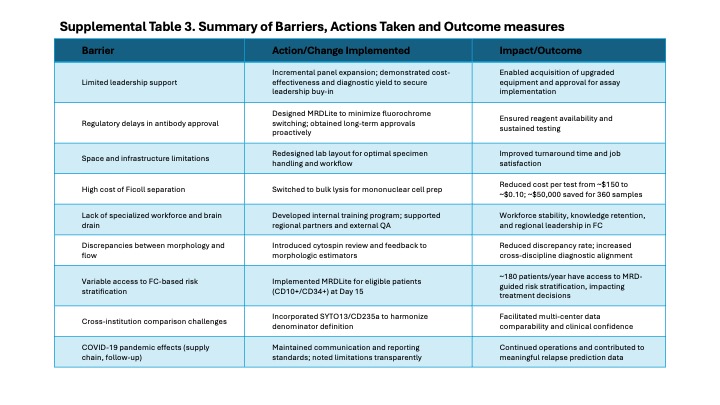

Supplement: Supplementary file 6 [file SupplementaryFile3.jpeg]
